# Pre-Ride Biomarkers and Endurance Horse Welfare: Analyzing the Impact of the Elimination of Superoxide Dismutase, δ-Aminolevulinic-Dehydratase, Thiobarbituric Acid Reactive Substances, Iron, and Serum Amyloid A Levels in Elite 160 km Endurance Rides

**DOI:** 10.3390/ani13101670

**Published:** 2023-05-17

**Authors:** Lena Bollinger, Alexander Bartel, Corinna Weber, Heidrun Gehlen

**Affiliations:** 1 Equine Clinic, Internal Medicine, Freie Universität Berlin, Oertzenweg 19b, 14193 Berlin, Germany; heidrun.gehlen@fu-berlin.de; 2 Institute for Veterinary Epidemiology and Biostatistics, Freie Universität Berlin, Königsweg 67, 14163 Berlin, Germany; alexander.bartel@fu-berlin.de; 3 Laboklin Veterinary Laboratory Diagnostics, Steubenstrasse 4, 97688 Bad Kissingen, Germany; dr_corinna_weber@gmx.net

**Keywords:** endurance horse, endurance riding, world championship, long-distance riding, superoxide dismutase (SOD), risk factor, elimination, iron, δ-aminolevulinic-dehydratase (ALAD), thiobarbituric acid reactive substances (TBARSs)

## Abstract

**Simple Summary:**

Endurance riding is a physiologically highly demanding equestrian sport for riders and athletes. The sport has strict vet controls; depending on the distance, between three and seven vet checks are performed to ensure that only healthy horses remain in the competition. Longer distances and higher physiological demands for the horse increase the risk of elimination due to lameness or metabolic reasons. Our study focuses on identifying a blood parameter—the enzyme superoxide dismutase—in order to maybe implement a blood test in the future to withdraw horses at risk of lameness before equestrian endurance events. The study population was still relatively small; therefore, further studies are necessary to validate the findings. We also measured a new parameter (ALAD) for the first time, although the results were not convincing.

**Abstract:**

High elimination rates and concerns for horse welfare are important issues in endurance riding. Improved understanding of the causes of elimination could increase completion rates in this sport. We have identified pre-ride laboratory risk factors that enable an assessment of potential elimination before the ride. A longitudinal cohort study was performed among 49 healthy horses competing in the 160 km endurance ride at the 2016 World Championship of Endurance Riding in Samorin/Slovakia. Blood samples were taken before the event. For statistical evaluation, horses were categorized into three groups: finishers, lame horses, and metabolically eliminated horses. Risk factors were calculated for each group using multinominal logistic regression. δ-Aminolevulinic-dehydratase (ALAD), thiobarbituric acid reactive substances (TBARSs), iron, and serum amyloid A (SAA) were measured and did not show an impact on the race outcome, but elevated pre-ride superoxide dismutase (SOD) was shown to have an effect on lameness elimination (*p* = 0.011). It might serve as an indicator for withdrawing horses at risk of later elimination before endurance rides, ultimately resulting in lower elimination rates and an increase in overall horse welfare.

## 1. Introduction

The demanding sport of endurance riding has come under scrutiny due to questions surrounding horse welfare and lethal injuries in the past decade [1,2,3,4,5]. After the disastrous World Equestrian Games in Tryon, USA, in 2018, the World Equestrian Federation (FEI) began seeking solutions to improve horse welfare, as well as the public perception of the sport [6].

Elimination rates at international rides have remained consistently high over recent ranging between 50% and 80%, while the speeds of the races have also increased: from 14.5 km/h in 2004 to around 18 km/h in 2015; the mean speed in the 2015 race was 19.2 km/h [4,7].

The purpose of this study was to identify pre-ride risk factors from acute-phase response (APR) and reactive oxygen species (ROS) in order to be able to identify horses at risk of elimination before a competition in the future. The listed parameters have partially been included in other prior studies, but never from an international championship with elite trained horses. Another purpose was to review the results obtained by Cywinska et al., both among a larger group of horses starting in a 160 km race and among elite international horse athletes [8]. Extreme running competitions are known to decrease the antioxidant capacity in humans; therefore, we hypothesized whether values of oxidative stress and SAA might serve as indicators for later elimination from elite endurance horses [9]. There has been scant research on endurance rides focusing on iron, and no relevant studies have considered iron values as pre-ride risk factors for elimination; thus, this paper addresses this gap through measurement of pre-ride iron levels [10].

Exercise induces an acute-phase response (APR) and enhances oxidative stress in several species such as horses, rats, and humans [11]. As a result of exercise, reactive oxygen species (ROS) are produced and need to be processed to prevent harm to the organism [12,13,14]. As ROS are difficult to measure due to their very short half-lives, products and co-factors of the different metabolic pathways need to be evaluated. The purpose of these different pathways is to prevent the lethal accumulation of free radicals in the organism. There is a wide variety of antioxidative substances and products of the reactions and lipid peroxidation. These substances include superoxide dismutase enzyme (SOD), thiobarbituric acid reactive substances (TBARSs), acute-phase protein serum amyloid A (SAA) as a health indicator, albumin as an anti-acute-phase protein, and δ-aminolevulinic-dehydratase (ALAD).

SOD levels, as measures of antioxidant capacity, have been shown to increase under prolonged training in humans. Equine serum SOD levels can also be increased through continuous exercise [15,16,17,18]. SOD in endurance horses has not yet been analyzed in detail, but has been part of some previous studies. Fraipoint et al. (2011) found significant differences in SOD levels pre- and post-exercise in endurance horses on a treadmill and in a field test. The study evaluated poorly performing horses in contrast to successful horses, and found lower SOD values post-exercise in poor performers [19,20]. The antioxidant status of endurance horses has already been studied [19,21,22,23].

Thiobarbituric acid reactive substances (TBARSs) are produced as a result of lipid peroxidation and have been studied in endurance horses before [19,24]. Holbrook et al. (2010) evaluated the pre-ride TBARS levels of 30 horses competing in 80 km or 160 km rides (15/15). They found the pre-ride values of TBARSs in six horses later eliminated to be higher than in the finishers of the 80 km competition. However, this was not the case for the eight eliminated horses in the 160 km ride. A potential reason discussed was the lower speed of 9.39 ± 1.26 km/h in the 160 km ride compared with 11.75 ± 1.91 in the 80 km ride. Holbrook et al. questioned whether TBARSs could serve as potential parameters indicating subsequent elimination [24]. Ott et al. (2022) found mildly elevated TBARS levels in moderately exercised horses as an adaptive response over a prolonged period of eight weeks [18]. ALAD, sometimes referred to as porphobilinogen synthase, was first identified in 1953. The enzyme catalyzes the reaction of 2 δ-aminolevulinic acid to porphobilinogen + 2 H_2_O and is involved in the heme biosynthetic pathway [25]. Lead has been demonstrated to inhibit the activity of ALAD; the simple natural process of red blood cell aging has also been shown to have an effect [26,27,28]. As a factor of heme synthesis, the inhibition of ALAD or its mutation may lead to anemia [29,30]. Using 5-aminolevulinic acid to treat equine sarcoids is described in a study by Golding et al., but there have not been any other equine studies on ALAD [30,31]. A study by Meine et al. (2022) showed the response of SOD and ALAD to injected high methionine concentrations in mice, confirming the findings of a similar experiment by Soares et al. (2017) in rats [31,32]. Interestingly, in both studies, the effect on ROS differed between organs such as the brain, liver, and kidney.

To the best of our knowledge, apart from our presented study, there have been no publications using the data of international endurance championships. This study sought to identify the measurable pre-ride blood parameters listed above pointing to later elimination, which could lead to lower elimination rates and increase animal welfare.

## 2. Materials and Methods

During the 2016 World Championship of Endurance Riding in Samorin (Samorin, Slovakia), blood samples were taken from 49 participating horses.

Participation in our study was voluntary and free of charge for the participants. Information on the study was sent to all starting national federations in advance via email, who then forwarded the information to their athletes. In addition, a social media post was shared to reach as many participants as possible. Owner-informed consent was obtained verbally before any animal examination or sampling proceeded. The study was performed in accordance with EU Directive 2010/63/EU, the German Animal Welfare Legislation, and the guidelines of Freie Universität Berlin on the protection of animals used for scientific purposes. Diagnostic blood collection is a common pre-ride procedure in elite equine athletes, and constitutes standard clinical veterinary practice. In accordance with the aforementioned legal regulations, this study could not be classified as an animal experiment. Sampling, therefore, did not require separate ethical approval according to German legislation, because it is usual practice to complete performance monitoring in endurance riding in accordance with good veterinary practice. The blood sampling was performed by trained veterinarians while the owners or legal representatives (FEI licensed and accredited trainers) were present.

### 2.1. Materials and Methods—Animals

The horses included in the study represented a total of 23 nations (Algeria, Australia, Bahrain, Belgium, Chile, Colombia, Croatia, Denmark, Ecuador, Germany, the United Kingdom, Hungary, Italy, Malaysia, the Netherlands, Norway, Portugal, Russia, South Africa, Sweden, Thailand, Uruguay, and the USA). Relevant information on the horses’ signalment (sex, age, breed, and color) was obtained from the FEI Horse Database. According to the FEI data, forty horses were purebred or crossbred Arabians. Four horses were listed as “other”, and five horses were referred to as “unknown”.

According to the official qualification criteria, all horses were at least eight years old and had successfully finished at least one 160 km *** (3 Star Ride—official FEI classification) ride at a minimum average speed of 14 km/h in the past 24 months, as well as two 120 km ** (2 Star Ride—official FEI classification) rides or higher in their career [8,21]. All horses were up to date on influenza vaccines, as required by the FEI.

During the race, the average air temperature was 18.9 °C with 83% humidity. The track was mostly flat with no significant hills. The weather was partly cloudy with repeating rain showers and moderate wind speeds.

The times between transportation, arrival at the venue, and blood sampling differed between the horses—ranging from five days before to the morning of the day before the race. All samples were drawn between 9:33 a.m. and 8:38 p.m.: 32.7% were obtained between 9 a.m. and 2 p.m.; 67.3% were obtained between 3 p.m. and 9 p.m. Before sampling, each horse underwent a general examination in accordance with the endurance rules, excluding gait. Heartrate, skin turgor, mucosal membrane, back and girth, gut sounds, and muscle turgor were recorded. Horses were not fastened during sampling. The horses were weighed using standardized weight measuring tape.

### 2.2. Materials and Methods—Sampling

In order to ensure that the study population was healthy, it was necessary to complement the sampling with a complete blood count and blood chemistry, including iron and albumin assessments.

Overall, 21 mL of jugular venous blood was collected on the left medial jugular vein, in accordance with normal protocols, using the Braun vacutainer^®^ system (Melsungen, Germany) with a 20-gauge-needle. Each horse was sampled using two 8.5 mL 16 mm × 100 mm PET hemogard serum tubes and one 4 mL 13 mm × 75 mm PET hemogard lithium heparin 17 IU/mL tubes.

### 2.3. Materials and Methods—Blood Chemical Analysis

Two laboratory technicians conducted blinded testing at the temporary laboratory set up at the venue. A lithium–heparin tube was used for the commercially available biochemistry panel in VetScanVS2 TM (Abaxis, Viernheim, Germany) with an Equine Profile rotor with full blood to directly measure albumin (ALB), blood urea nitrogen (BUN), creatinine (CREA), gamma-glutamyl transferase (GGT), aspartate-aminotransferase (AST), globulin (GLO2), glucose (GLU), and total bilirubin (TBIL) at the venue. The results were transferred from the laboratory machines directly into Scil VIP Manager Software (Viernheim, Germany). According to Abaxis requirements, lithium–heparin was spun off at 4000 g/4 min if hematocrit levels were >50%; then, plasma was used for the Equine Profile Rotor. If hematocrit levels were <50%, full blood was used.

### 2.4. Material and Methods—SOD, TBARS, SAA, Iron, and ALAD Evaluations

Serum samples were frozen directly after being spun off and were transported to Laboklin Lab Bad Kissingen (Bad Kissingen, Germany) for the sampling of SOD, TBARSs, SAA, iron, and ALAD. A licensed veterinarian performed the blinded testing at Laboklin Lab. To assess SOD, an assay kit from Cayman Chemicals was used, measuring all three types of iso-enzymes (Cu/Zn, MN, and FeSOD) as one combined value. SOD in horses is influenced by age, breed, sex, and exercise [13,33,34,35,36,37]. Oral supplements such as vitamin E, SOD, or polyunsaturated oil have been shown to have an effect [34,38,39,40], but an increased selenium intake does not affect SOD levels [11,15]. SOD was shown to be altered by methionine injections in rats and mice [32,41]. Iron and SAA were tested with a Cobas 8000 instrument acquired from Roche. TBARS and ALAD assay kits from Bio-Techne were used.

Horses were divided into three groups: finishing (18 horses), lame (20 horses), and metabolically eliminated (11 horses) athletes. All blood values were checked for normality using histograms compared against prior knowledge about distribution. SOD, TBARSs, SAA, iron, and ALAD levels had to be log_2_-transformed to achieve a normal distribution. For the statistical evaluation, we used multinomial logistic regression with finishing as reference versus lameness or metabolic elimination (R Package nnet version 1.8–31 [42]). We calculated univariable odds ratios with 95% confidence intervals.

### 2.5. Material and Methods—Statistical Analysis

All possible confounders were identified using R Package DAGitty software [43] and checked for association with exposure and outcome. Multivariable analyses were adjusted for the identified confounders: age, sex, and weight. For all confounders, crude odds ratios are reported. All *p*-values ≤ 0.05 were considered to be significant. All statistical analyses were performed using R Version 4.0.0 (R Foundation Vienna). Breed was not considered as a confounder because the variable “breed” only had a weak association with the race result (Cramer’s V = 0.353); most horses were either purebred or crossbred Arabians.

### 2.6. Limitations

Due to the ride studied here being a championship, the atmosphere may have been more competitive, and therefore may have had encouraged riders to push the horses further than in normal rides [44].

Sampling only took place before one race, multiple parameters were evaluated, and there were only 49 participating horses, which must be considered as limitations of this observational study. In addition, sampling times among the analyzed horses were not standardized because riders were not willing to change their regular routines due to the ride being a competitive championship. Additionally, evaluation of the frozen samples took place six months after the ride. It is not known if the storage time affected the measured values; however, this time frame was the same for all samples.

## 3. Results

In total, 131 riders started in race; 47 riders completed it. This represents a completion rate of 35.87%, which is higher than finishing rates of the world championships which took place in 2014 (22.89%) and 2012 (35.37%) [45]. No data for 2018 are available because the ride was canceled.

Regarding the total race, 21 horses (16.03%) were eliminated for metabolic reasons, 4 riders retired (3.05%), 3 riders failed to complete a loop (2.29%), of whom 1 suffered a catastrophic injury, and 56 horses (42.74%) were eliminated due to lameness.

A total of 49 horses were included in the study. None of the horses exhibited abnormalities in the pre-sampling examination, and all passed the pre-ride inspection. Of the 49 horses included in this study (23 mares/26 geldings), 11 horses (22.45%) were eliminated due to metabolic issues, 20 horses (40.82%) were eliminated due to lameness issues, and 18 horses (36.73%) finished the race.

**Figure 1:**
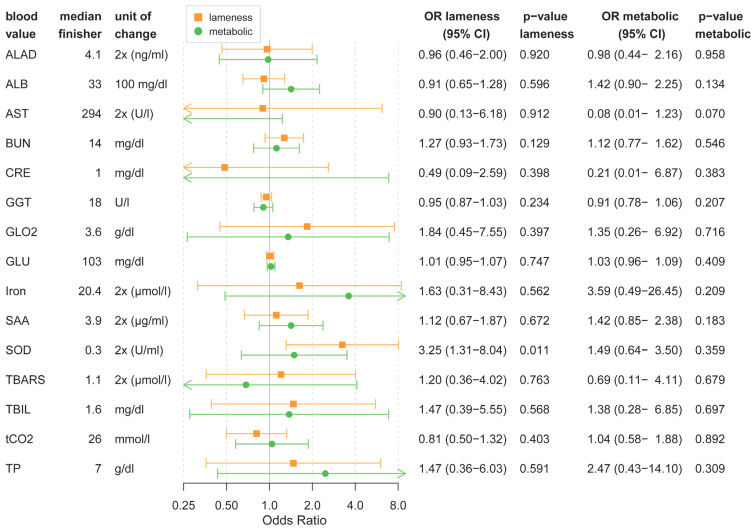
Forest plot of odds ratios (ORs) for lameness or metabolic elimination depending on pre-race blood values. OR values close to 1 meant that the blood value had no influence on elimination. OR > 1 meant that higher blood values increased the odds of elimination; OR < 1 meant that lower blood values increased the odds of elimination. Median blood values of finishers are provided as a comparison. *p*-values ≥ 0.05 were considered significant.

All variables presented in the following section have been adjusted for age, sex, and weight. Only small changes in blood values from regular blood chemistry tested with VetScan2 were observed. None of the findings indicated a pathological process. The reference values for all following parameters were the median values finisher horses, and are presented in Figure 1.

For every doubling of the SOD value, there was a 3.25-fold higher chance of lameness elimination (CI 1.31–8.04, *p* = 0.011). The effect was much smaller regarding metabolic elimination (OR 1.49, CI 0.6–3.5, *p* = 0.359).

The *p*-values for the following parameters for elimination were not significant, but the overall number of samples in individual endurance races is always comparatively low, making the world championship with 49 horses already one of the larger study populations. Regarding statistical basic knowledge, using *p*-values as the only statistical marker to evaluate results in endurance publications might lead to the exclusion of clinically relevant information. Therefore, the following results are presented despite some *p*-values being greater than 0.05 and are compared with the existing literature in the Discussion section.

TBARSs had no effect on the chance of any elimination. Similarly to other authors, we also found a wide range of individual TBARS values (see Table 1). Higher SAA levels showed no effect on lameness elimination (OR = 1.12, CI 0.67–1.87, *p* = 0.672) and resulted in a small increase in the chance of metabolic elimination (OR = 1.42, CI 0.85–2.38, *p* = 0.183), but these effects did not cross the significance threshold. Additionally, the SAA medians and ranges were 3.9 (3.5–39.0) in finisher horses, 3.9 (3.5–233.7) in lame horses, and 4.4 (3.5–379.4) in horses eliminated for metabolic reasons.

Horses showing an elevation of Albumin before the competition had a 1.42-fold higher chance of elimination for metabolic reasons (CI 0.90–2.25, *p* = 0.134) and a lower chance of elimination due to lameness (OR = 0.91, CI 0.65–1.28, *p* = 0.596).

For the first time, ALAD was measured in horses, showing a lameness *p*-value = 0.920 (OR = 0.96) and metabolic *p*-value = 0.958 (OR = 0.98). Confidence intervals in horses eliminated due to lameness showed a range of CI 0.46–2.0, whereas confidence intervals in horses eliminated for metabolic reasons showed a range of CI 0.44–2.16.

Overall, ALAD showed no effect on elimination.

Higher iron levels showed an increase in the chance of elimination, but again, these effects did not cross the significance threshold, and the width of the confidence intervals indicated a high uncertainty about the real effect.

The effect of pre-race blood values on elimination is shown in Table 1.

## 4. Discussion

This study analyzed the race results and blood parameters of 49 horses competing in the 2016 World Championship of Endurance Riding in Samorin, Slovakia. To the best of our knowledge, this is the first time that blood samples of horses competing in an international endurance championship have been analyzed. It was hypothesized that different blood values prior to the ride would affect the chance of elimination and were expected to indicate differences among blood samples of the finishers, lame, and metabolically eliminated horses in parameters associated with oxidative stress and acute-phase response. The study showed that elevated pre-ride values of SOD had a negative impact on the outcome of the race regarding elimination due to lameness.

Regarding TBARSs, there were no differences between finishers and eliminated horses, regardless of the cause of elimination. The mean speed in the race (19.2 km/h) and in the study population (18.6 km/h) was considerably higher than in the study conducted by Holbrook et al. [24]; therefore, we conclude that higher pre-ride TBARS values in horses eliminated from an 80 km competition are not related to the higher speed of the ride. As this has been shown to be the case in humans, we instead conclude that horses competing in 160 km rides, having been trained for years, have metabolisms adapted to long endurance exercise. With proper training, the oxidant capacity is known to improve [9]. It seems plausible that elite horses competing in the world championship are at the highest possible training level, demonstrating an optimal oxidant capacity. Exploring how the oxidative capacity develops during training and over increasing ride distances of 80, 120, and 160 km might be the starting point of further studies.

For SAA, there were no differences in pre-ride values between finishers and eliminated horses. This contradicts the findings of Cywinska et al. [8]. Equine serum amyloid A has been studied widely, also being the subject of a limited number of studies on endurance horses [8,46,47,48,49]. In one study, pre-ride SAA levels in five short-distance (up to 80 km), inexperienced horses were higher than in seven long-distance (120–160 km), experienced horses [48]. In another study by the same author, no differences in the pre-ride values in 17 finisher horses of different ride lengths could be observed [47]. Only one study evaluated SAA levels in long-distance (120 km or more) horses as a marker for later elimination. Cywinska et al. (2010) sampled endurance horses competing in 120 and 160 km races. Pre-ride SAA values in horses that ended up being eliminated were higher than in finisher horses (5809.522427 ng/mL vs. 411.7144 ng/mL). However, the ratio between horses eliminated due to lameness (9 horses) and metabolic reasons (3 horses) was uneven, and the overall numbers of horses (20 horses) and starters in each ride length (10/10) were comparatively low. During training, not only does acute-phase protein production (such as SAA) change; cytokine production is also altered, leading to the creation of an anti-inflammatory state in horses trained to compete in endurance races. Referring to Witowska et al. (2019), a positive modulation in the cytokine anti-inflammatory state can be seen after 8 to 12 weeks of training [50]. Additionally, lower ROS levels and an anti-inflammatory cytokine state occur in well-trained race horses [51]. According to FEI rules, horses competing in the world championship must have already completed numerous long-distance endurance rides, and therefore have been training for several years; thus, it seems plausible that the adaption of resting SAA levels and protective cytokine production is similar to resting CRP (C-reactive protein) values in humans [52,53]. Concluding that pre-ride SAA levels might serve as an indicator for elimination in inexperienced short- to middle-distance endurance horses, they should not be taken into consideration when attempting to predict the elimination risk in experienced long-distance horses trained for elite endurance races.

Albumin provides the highest percentage of total protein; thus, the conclusion can be drawn that albumin levels, similarly to total protein, decrease during an endurance season [54]. This is likely linked to exercise-induced hypovolemia in horses, also representing an adaption process to prolonged exercise [55]. All horses included in our study showed no pathological abnormalities in bloodwork and clinical examination before the ride; thus, we defined our study population as healthy. Therefore, we assume that individual hyperalbuminemia and slightly increased ORs for metabolic elimination are the result of dehydration (increased hematocrit). As shown in a previous report on the same results of the 2016 World Championship, hematocrit was associated with a 1.26-fold higher risk of lameness, and even a 1.4-fold higher risk of elimination due to metabolic reasons [56]. As muscular activity produces heat, the body cools itself down by producing sweat. Losing water, the body dehydrates, which reduces blood flow in the muscles and may lead to lameness, and later even more and severe metabolic and potentially life-threatening problems [57]. Potential reasons for pre-ride hypovolemia have been discussed in detail in our previous study, identifying transportation as one of the causes. The observed hyperalbuminemia in metabolic horses may support this theory; Padalino et al. found albumin to be elevated after transportation [58]. Reduced perfusion and resulting transportation has been identified as a negative parameter of inflammation; synthesis decreases with the increase in globulins [59,60]. Total protein levels increase over the duration of an endurance ride [61]. In a 2019 study, albumin was shown to increase as a result of slow, long-lasting exercise compared with short, intense exercise [62].

Iron values could not be used as an indicator for potential race elimination in this study. None of the values exceeded common equine reference values, which was not surprising because the horses in the study population were highly trained athletes with a very low disposition to diseases and hemorrhages [63].

Iron in horses is not nearly as relevant as in humans—mainly due to the rare occurrence of iron deficiency in equines [10,64,65,66,67,68,69,70,71]. Serum iron concentrations have also been studied as a marker in systemic inflammatory diseases [72,73,74]. During prolonged exercise, the iron binding antioxidant activity decreases and iron levels in plasma may increase [75]. Even though it is fairly common to supplement iron in horse athletes, there is no contemporary scientific evidence proving that this is necessary or beneficial [69]. In fact, there is even a risk of iron overload due to supplemental feeding [67,76,77]. Moreover, only a few publications related to endurance riding have used confounder adjustment during statistical evaluations [78,79,80]. Elimination risk seems to be a result of multiple factors; therefore, confounder adjustment is indispensable. Other risk factors not included in confounder adjustment may include venue- and environment-related aspects, riding strategy, age, or high running speeds [44,80,81,82].

According to our findings, ALAD had no influence on the outcome of the race. As ALAD has not previously been studied in horses and there are currently no available reference values, the results presented here might serve as an initial evaluation of ALAD values in equines. The role of ALAD in athletes has not been examined until now, either in humans or in equines. However, because ALAD has previously been linked to oxidative stress in human diseases, this study evaluated ALAD in a healthy horse population for the first time [83,84].

In this study, lower pre-ride SOD values were beneficial to the race outcome. It is possible that SOD is only altered after prolonged exercise, such as endurance rides over 120 km long [11]. This matches the conclusion of Kinnunen et al. (2005), who identified general resting oxidative stress markers (oxygen radical absorbing capacity, vitamin E, and lipid hydroperoxide (LPO) concentrations or glutathione-related enzyme activities) in endurance horses to be higher than in trotters. However, prolonged exercise of an 80 km ride did not induce oxidative stress in endurance horses [23]. It was expected that higher pre-ride SOD values would prevent subsequent elimination, but in fact, the opposite was found. SOD is known to increase after prolonged exercise; therefore, we conclude that horses with higher SOD levels at the time of sampling had been exposed to longer-lasting oxidative stress of unknown origin, possibly resulting from over-training or insufficient rest to recover from exercise [85,86]. Therefore, the oxidative capacity for the upcoming race had already been lowered, with SOD levels in the horses’ blood representing current SOD activity and oxidative stress defense, but not representing SOD storage. After a race, there is less SOD available to prevent oxidative damage; therefore, SOD values were lower in eliminated horses than in successful horses. Further studies to confirm this hypothesis, possibly with a muscle biopsy to evaluate muscle SOD before the ride, could provide more detailed insights. As there are no precise numbers on pre-ride or resting SOD values in endurance horses available, besides the few horses studied by Fraipoint et al. (2011), it is not known which numbers reflect a healthy resting state and which values could point to increased SOD activity in current oxidative stress. Our test evaluated overall SOD levels in plasma. We therefore cannot make any statement on the distribution of the three existing isoenzyme types which, in humans, are affected differently depending on the kind of exercise.

## 5. Conclusions

The theory concerning lower pre-ride SOD levels may be supported by the findings of Fraipoint et al. [40]. Over a three-month testing period, SOD values in poorly performing endurance horses were shown to be lower after exercise compared with successful horses. In summary, regarding the results of both studies, we assume the following: horses with a lower pre-ride SOD value have a greater SOD capacity left to mobilize in order to cope with oxidative stress and ROS. SOD values after a race in finisher horses are higher as a result of oxidative stress defense. Horses with a higher pre-ride SOD value have already activated their SOD capacity for unknown reasons. As a result of our findings, we conclude that SOD might serve as a pre-ride indicator for subsequent elimination during endurance races. To validate these findings, further studies on different race lengths with more subjects and horses are necessary.

## Figures and Tables

**Table 1:** Distributional characteristics of the study population and summary statistics of blood values.

			Finished	Lameness	Metabolic	Missing
*n*			18	20	11	
Sex (%)						
	Male		8 (44.4)	11 (55.0)	7 (63.6)	0%
	Female		10 (55.6)	9 (45.0)	4 (36.4)	
Age (median [range])		years	12.0 [9.0, 16.0]	11.0 [8.0, 17.0]	11.0 [8.0, 17.0]	0%
Weight (median [range])		kg	405.0 [345.0, 440.0]	402.0 [350.0, 450.0]	408.0 [380.0, 450.0]	0%
Time between blood sampling and race (mean [SD])		days	2.8 (1.1)	2.7 (0.9)	3.0 (0.8)	0%
Breed (%)						
	Purebred Arabians		8 (44.4)	10 (50.0)	6 (54.5)	0%
	Anglo-Arabians		2 (11.1)	5 (25.0)	0 (0.0)	
	Shagya-Arabians		2 (11.1)	2 (10.0)	0 (0.0)	
	Partbred Arabian		1 (5.6)	0 (0.0)	3 (27.3)	
	Other		2 (11.1)	1 (5.0)	1 (9.1)	
	Unknown		3 (16.7)	2 (10.0)	1 (9.1)	
Median [range]						
ALAD		ng/mL	4.1 [2.1, 31.0]	3.6 [1.9, 33.6]	4.0 [2.1, 16.0]	12.2%
ALB		100 mg/dL	33.0 [30.0, 35.0]	32.5 [29.0, 38.0]	34.0 [31.0, 39.0]	0%
AST		U/L	294.0 [198.0, 623.0]	307.5 [204.0, 520.0]	283.0 [185.0, 357.0]	2%
BUN		mg/dL	14.0 [10.0, 16.0]	14.5 [10.0, 22.0]	13.0 [11.0, 17.0]	0%
CRE		mg/dL	1.0 [0.6, 4.2]	1.1 [0.8, 1.2]	1.1 [0.6, 1.4]	0%
GGT		U/L	18.0 [11.0, 63.0]	16.5 [12.0, 43.0]	16.0 [11.0, 23.0]	0%
GLO2		g/dL	3.6 [2.3, 4.8]	3.4 [3.3, 4.9]	3.5 [3.0, 4.2]	0%
GLU		mg/dL	103.0 [81.0, 130.0]	100.5 [88.0, 135.0]	105.0 [96.0, 166.0]	0%
Iron		µmol/L	20.4 [6.4, 31.8]	19.7 [10.4, 47.2]	25.4 [11.3, 30.1]	14.3%
SAA		µg/mL	3.9 [3.5, 39.0]	3.9 [3.5, 233.7]	4.4 [3.5, 379.4]	2%
SOD		U/mL	0.3 [0.1, 1.1]	0.8 [0.2, 9.0]	0.6 [0.1, 1.7]	10.2%
TBARSs		µmol/L	1.1 [0.8, 9.1]	1.1 [1.0, 3.8]	1.1 [0.8, 1.9]	10.2%
TBIL		mg/dL	1.6 [1.2, 3.1]	1.6 [1.1, 3.2]	1.7 [1.2, 2.6]	0%
TP		g/dL	7.0 [5.4, 8.0]	7.0 [6.3, 8.0]	6.9 [6.5, 7.6]	0%

ALAD, δ-aminolevulinic-dehydratase; ALB, albumin; AST, aspartate-aminotransferase; BUN, urea; CRE, blood creatinine; GGT, Gammaglutamyl-Transferase; GLO2, globulins; GLU, glucose; Iron, iron; SAA, serum amyloid A; SOD, superoxide dismutase; TBARSs, thiobarbituric acid reactive substances; TBIL, total bilirubin; TP, total serum protein.

## Data Availability

Aggregate participant data that underlie the results are available from the corresponding author upon reasonable request. Results data are available from the corresponding author and online at: https://inside.fei.org/fei/disc/endurance/main-events/endurance-past (accessed on 11 April 2023).
